# Timing of preoperative antibiotics for knee arthroplasties: Improving the routines in Sweden

**DOI:** 10.1186/1754-9493-5-22

**Published:** 2011-09-19

**Authors:** Annette W-Dahl, Otto Robertsson, Anna Stefánsdóttir, Pelle Gustafson, Lars Lidgren

**Affiliations:** 1 The Swedish Knee Arthroplasty Register, Department of Orthopedics Skåne University Hospital Lund, 221 85 Lund, Sweden; 2 Department of Orthopedics, Clinical Sciences Lund, Lund University, 221 85 Lund, Sweden; 3 Department of Orthopedics, Skåne University Hospital Lund, 221 85 Lund, Sweden

**Keywords:** Knee arthroplasty, Infection prophylaxis, Prophylactic antibiotics, Preoperative routines

## Abstract

**Background:**

A slight increase in revisions for infected joint arthroplasties has been observed in the Nordic countries since 2000 for which the reasons are unclear. However, in 2007 a Swedish study of the timing for prophylactic antibiotics in a random sample of knee arthroplasties found that only 57% of the patients had received the antibiotic during the optimal time interval 45-15 minutes before surgery. The purpose of the report was to evaluate the effect of measures taken to improve the timing of prophylactic antibiotics.

**Findings:**

Reporting this finding to surgeons at national meetings during 2008 the Swedish Knee Arthroplasty Register (SKAR) introduced a new report form from January 2009 including the time for administration of preoperative antibiotics. Furthermore, the WHO's surgical checklist was introduced during 2009 and a national project was started to reduce infections in arthroplasty surgery (PRISS). The effect of these measures was found to be positive showing that in 2009, 69% of the 12,707 primary knee arthroplasties were reported to have received the prophylaxis within the 45-15 min time interval and 79% of the first 7,000 knee arthroplasties in 2010. A survey concerning the use of the WHO checklist at Swedish hospitals showed that 73 of 75 clinics had introduced a surgical checklist.

**Conclusions:**

By registration and bringing back information to surgeons on the state of infection prophylaxis in combination with the introduction of the WHO checklist and the preventive work done by the PRISS project, the timing of preoperative prophylactic antibiotics in knee arthroplasty surgery was clearly improved.

## Background

During a meeting in 2007 of the Nordic Arthroplasty Registers (NARA, Denmark, Norway and Sweden) it was reported that after the year 2000 there were signs of an increase in the number of infected revisions after hip arthroplasty. Similar trends have since been observed after knee arthroplasty in Finland [1], Norway [2] and Sweden [3].

**Figure 1:**
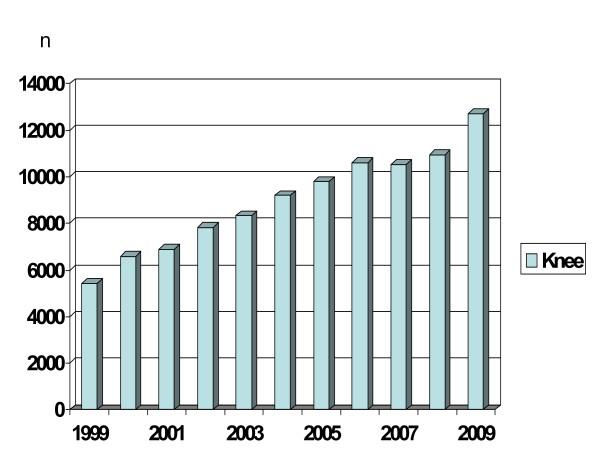
**Primary knee arthroplasty 1999-2009 in Sweden**.

At the time when arthroplasty was introduced in the Nordic countries the surgery was considered highly specialized and obeyed to strict antiseptic and aseptic routines. However, the surgery quickly became popular, and during the last decade the number of knee arthroplasty procedures more than doubled (Figure 1). The "industrialization" of the arthroplasty surgery has probably made it increasingly difficult to constantly maintain important preventive measures and the high initial standards.

### Infection prophylaxis

Use of prophylactic antibiotic is an effective measure to reduce the risk of infection in joint arthroplasty surgery [4]. To ensure that there is an adequate antibiotic concentration in the tissues at surgery, the timing of preoperative prophylaxis is crucial [5].

The aim of preoperative systemic prophylactic antibiotics is to have the concentration in the tissues at its highest at the start and during surgery. For the most commonly used antibiotics, it has been considered optimal to administer the drug intravenously 30 min before skin incision [6,7] and it has been documented that administration more than 60 min preoperatively is associated with higher risk of surgical infection [8]. Common antibiotics for joint arthroplasty surgery such as the beta-lactam derivate cloxacillin and cephalosporin have a short half-life. When using a tourniquet, which often is the case in knee surgery, it is important that such antibiotics are not administrated to late if a reasonable concentration is to be reached in the tissues [9]. Thus it is recommended that the infusion should be given 45-15 minutes before start of knee arthroplasty surgery.

In a study comparing two different doses of prophylactic antibiotics in patients having high tibial osteotomy for osteoarthritis we found that one third of the patients had not received their prophylactic antibiotics at all or only received it postoperatively [10]. Further, a small study at our own university clinic in Lund, initiated by a strategic program against antibiotic resistance (STRAMA), indicated that the timing of prophylactic antibiotics was inadequate.

### Inadequate timing of prophylactic antibiotics in orthopedic surgery

This indication of suboptimal routine resulted in a more detailed study regarding the timing of preoperative antibiotic administration at our own clinic in Lund as well as of a randomized sample of knee arthroplasties reported to the Swedish Knee Arthroplasty Register (SKAR) in 2007 [5].

We found that of 114 orthopedic patients in Lund during 2008, only 45% (95% CI: 36-54%) received their first antibiotic dose 45-15 min before the start of surgery. In 22 cases (19%), surgery was started at the same time or before administration of the antibiotic. In the national randomized sample from SKAR in 2007 57% (CI: 50-64%) received the antibiotic 45-15 min before the start of surgery and 53% (CI: 46-61%) 15-45 min before the tourniquet was applied [5].

In the present short report, we report the results from the SKAR of measures taken to improve the timing of prophylactic antibiotics.

## How to improve

### Information back to the units

Our findings showed that the present situation regarding the timing of preoperative prophylactic antibiotics was inadequate and that the routines needed improvement [5]. The study was presented at the annual national knee and hip arthroplasty register meeting in November 2008 and to the heads of departments at the annual orthopaedic society meeting.

### New form for registration of knee arthroplasty surgery-on a national level

At the annual register meeting in 2008 it was decided that the SKAR should start using a new extended report form for knee arthroplasties that included information on the timing of preoperative antibiotic administration; if prophylactic antibiotics was used (yes/no), if yes, the name of the antibiotics, dose and number of doses per 24 hour, start preoperatively (yes/no), if yes, minutes before start of surgery and planned length of treatment (days). The new form was tested, evaluated and revised during 2008 and started fully January 2009.

The SKAR was initiated in 1975 when Swedish surgeons realized that at this time of rapid development it would be impossible for an individual surgeon to base his choice of optimal operative treatment on his own experience and the literature [11]. All hospitals performing knee arthroplasties in Sweden reporting the primary as well as the revision procedures to the SKAR. The coverage of the SKAR is 100% and the completeness is 97% [3]. The report form include information on the unique personal number including information on age and gender, diagnosis, surgical date, prosthesis components inserted including article and LOT numbers, cemented/uncemented, type of cement and since January 1^st ^2009 also surgical techniques and prophylactic treatments.

An English version of the annual reports including the new form is available on http://www.knee.se.

### WHO's surgical safety checklist

At the same meeting SKAR representatives suggested that the orthopedic clinics should start to use the checklist for surgery, initiated from WHO, with a standard "time-out" before skin incision, during which the administration of prophylactic antibiotics was confirmed [12].

The checklist was translated into Swedish and available in March 2009 and gradually introduced and adapted by a work group including representatives from surgery, orthopedics, anaesthesiology, operating and anaesthesiology nurses [13].

### The PRISS project

From 2009 a project "Prosthetic related infections should be stopped" (PRISS) started in a collaboration of the national professional associations of orthopaedic surgeons, infection specialists and orthopaedic nurses. It is supported by the Patient Insurance (LÖF) [13] as prosthetic related infections constitute a significant part of the insurance company's cases.

The purpose of the project is to improve the routines of prevention of infections in arthroplasty surgery and decrease the infection rate with 50%. Taking part in the project is voluntary. However during the spring 2011 80% (60/76) of the clinics performing knee and hip arthroplasties in Sweden (public as well as private) were participating in the project.

### Statistics

The 95% confidence interval (CI) for proportion was calculated as p_s _± 1.96 times the standard error of p_s_, where p_s _is the proportion of patients receiving prophylactic antibiotic in adequate time.

## Results

The new SKAR register form has been in use since January 1st, 2009. Although it takes time to adapt to new routines the reporting during the first year was better than expected (information was missing for 11%). 80% of the units reported that Cloxacillin was the antibiotic used in 80% of cases (12,707 patients). The most common dose was 2 g × 3. The planned length of treatment was most often 24 hours although this varied from 8 hours to 48 hours. Clindamycin was used in 5.9% of the surgeries which can be interpreted as the percentage of the patients being suspected of having penicillin allergy.

**Figure 2:**
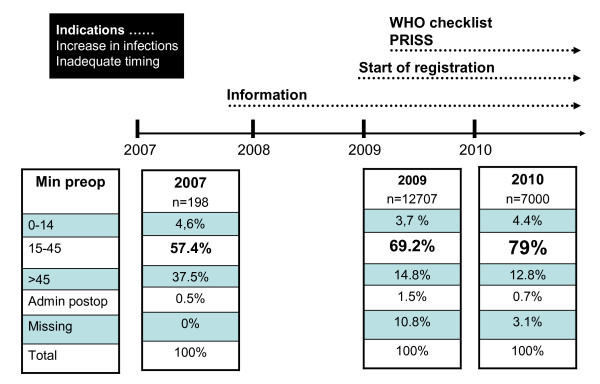
**Flow-chart of interventions and results of the timing of prophylactic antibiotics in knee arthroplasties in Sweden**.

During 2009, 69% (CI: 68-70%) of the 12,707 primary knee arthroplasties were reported to have received their prophylactic antibiotics within the 45-15 min. time interval [3] (Figure 2).

Corresponding figures for 2010 according to the timing of prophylactic antibiotics showed further improvement with 79% (CI: 78-80%) having the prophylactic antibiotic within the 45-15 minutes time interval (Figure 2).

In February and March 2011 the SKAR sent out a survey asking if the clinics that performed knee arthroplasties used a surgical checklist. All 75 clinics answered the survey. All except two clinics used a surgical checklist, 35 of 73 clinics used the entire WHO surgical checklist and the rest a modified form. 60 of the 73 clinics had started using the checklist by 2009.

## Discussion

The increase in number of patients receiving their prophylactic antibiotic within 15-45 minutes prior to arthroplasty surgery suggests that routines have improved probably as the result the time of administration being registered in combination with the introduction of the WHO checklist with a "time-out" and the extensive preventive work done by the PRISS project.

The revision rate due to infection in primary knee replacements is around 1% and 0.3-0.6% in hip replacements [1,14,15]. However, we know that these figures are an underestimation as the revision rate does not truly reflect the infection burden [1].

It will probably take 3-5 years to show a decrease in the risk or infection after knee arthroplasty (13 000 primary knee arthroplasties are carried out yearly). However, the goal in Sweden is 50% reduction in infections after knee arthroplasty by improvement in the routines for infection prophylaxis. With an estimated total infection rate of 1.5% in knee arthroplasty during the first two postoperative years, a decrease of 50% should not only result in savings of at least 4 million Euros yearly but as well, even more important, save the patients from unnecessary suffering.

## Conclusion

By information on outcome to the operating surgeons and national registration in combination with the introduction of the WHO checklist and the preventive work done by the PRISS project, the timing of preoperative prophylactic antibiotics in knee arthroplasty surgery was improved. To be able to show that these interventions decrease infections after knee arthroplasties will take some more years.

## Competing interests

The authors declare that they have no competing interests.

## Authors' contributions

AWD and LL were developing the idea of this study. AWD, OR, AS, PG and LL contributed to the study design. AWD and OR collected and analysed the data from the SKAR. All authors prepared the manuscript. All authors read and approved the final manuscript.
